# Editorial: Hemostasis and Stroke

**DOI:** 10.3389/fneur.2021.737556

**Published:** 2021-08-11

**Authors:** Zsuzsa Bagoly, Daniel Behme, Johannes Kaesmacher, Sara Martinez De Lizarrondo

**Affiliations:** ^1^Division of Clinical Laboratory Sciences, Department of Laboratory Medicine, Faculty of Medicine, University of Debrecen and Eötvös Loránd Research Network-University of Debrecen Cerebrovascular and Neurodegenerative Research Group, Debrecen, Hungary; ^2^Faculty of Medicine, Otto von Guericke University Magdeburg, Madgeburg, Germany; ^3^University Institute of Diagnostic and Interventional Neuroradiology and University Institute of Diagnostic, Pediatric and Interventional Radiology, University Hospital Bern, Inselspital, University of Bern, Bern, Switzerland; ^4^INSERM UMR-S U1237, Physiopathology and Imaging of Neurological Disorders, GIP Cyceron, Institut Blood and Brain @ Caen-Normandie (BB@C), Bd H. Becquerel, BP 5229, Caen, France

**Keywords:** hemostasis, stroke, thrombolysis, hemorrhage, thrombophilia, outcome, risk factors

Stroke is the leading cause of death and permanent disability worldwide (1, 2). Understanding the hemostasis system in stroke presents an exciting and relatively new area of research with potential applications in stroke therapy (3–5). Better understanding of the alterations of the hemostasis system, particularly its counterplay with inflammation and vascular biology has tremendous potentials in stroke management. In this Special Issue, original research articles and reviews focus on most recent advances on hemostasis, thrombosis or vascular biology related to all areas of stroke research.

Hemorrhagic transformation (HT) after ischemic stroke is a common and potentially severe complication (6). HT carries a high risk of clinical deterioration and is associated with poor outcomes and mortality (7). Early recognition or prediction of HT would be an important aid for the appropriate management of AIS patients. Due to the importance of this topic, it is not surprising that a series of papers in this Special Issue aimed to improve our understanding of HT. In the research paper by He et al. the authors investigated whether an easily accessible and rapid screening tool, the neutrophil-platelet ratio (NPR) could predict HT in patients with AIS. In theory, NPR shows its advantage in revealing information about the crosstalk between inflammation and hemostasis. Based on a cohort of 279 stroke patients, the authors conclude that high NPR (>39.9) was independently associated with the increased risk of HT, particularly that of parenchymal hematoma in AIS (OR = 2.00, 95%CI: 1.041–3.843, *p* = 0.037). Not only low platelet count, but impaired platelet function may also increase the risk of HT. Rapid testing of platelet count is a well-established standard in the treatment of AIS and patients with low platelet counts are excluded from recombinant tissue plasminogen activator (rt-PA) treatment due to the significantly increased risk of HT (8). Although current guidelines do not recommend it, whether platelet function testing could be used to guide treatment decision during the acute phase of AIS is unknown (9). In the study by Lieschke et al. the authors aimed to demonstrate the feasibility of incorporating flow cytometry-based platelet function testing in a mouse model of dual antiplatelet therapy followed by ischemic stroke and rt-PA infusion (Yuan et al.). In addition to monitoring the efficacy of antiplatelet therapy, they sought to investigate the impact of platelet function in the development of HT. Their results suggest that reduced platelet activation is indicative of an increased risk for HT following experimental stroke and rt-PA treatment. Another important factor that might contribute to HT in AIS patients could be the impaired hemostasis balance due to liver fibrosis. The paper by Yuan et al. highlights that although subclinical liver fibrosis or steatosis may not be rare in patients with stroke, data are lacking regarding the association between liver fibrosis and HT for patients with AIS (10). In their single center retrospective study, 185 consecutive patients with HT and 199 age- and sex-matched AIS patients without HT were enrolled, and the extent of liver fibrosis was assessed using a validated fibrosis index (FIB-4). After adjustments for potential confounders, the authors concluded that AIS patients with a high FIB-4 score had a 3.461-fold risk (95%CI: 1.404–8.531) of HT as compared to patients with low FIB-4 score. HT may be a rare but severe complication of adult ischemic Moyamoya disease (MMD) (11). In the research paper by Lu et al. the authors investigated the differences of clinical and radiological features between adult ischemic MMD patients with and without HT and studied clinical outcomes. Their data suggest that normal cerebral perfusion may be a risk factor associated with HT in adult ischemic MMD. As expected, HT was strongly associated with increased disability rates and mortality in the investigated cohort of patients.

HT following AIS may be facilitated by rt-PA therapy. rt-PA has been the mainstay of therapeutic thrombolysis in AIS, however, in ~6–8% of treated patients, potentially devastating intracerebral hemorrhage may occur (12). Moreover, rt-PA may have other effects on the central nervous system by modulating the blood brain barrier (BBB) permeability and influencing neuroinflammatory processes (13). These include the complement system that has received much attention in CNS disorders in recent years. While the role of the complement's cascade in neuroinflammation, neurodegenerative disease, and CNS injury is well-recognized, its role in rt-PA mediated neuroinflammation and stroke has not been fully explored as yet. Tenecteplase (TNK-tPA) is a challenging alternative of rt-PA, developed over 25 years ago to circumvent the short half-life of t-PA (14). Parallel studies on TNK-tPA is important in studies attempting to investigate the efficacy and safety of rt-PA. In their original work, Keragala et al. demonstrate that inhibition of the complement C5a-C5R1 interaction reduces the ability of rt-PA and TNK-r-PA to increase BBB permeability, which may offer novel means to improve the safety profile of thrombolytic therapy for patients with AIS.

HT and the increase of BBB permeability is not the only concern in patients receiving thrombolytic agents. Unfortunately, the majority of patients who receive thrombolysis using rt-PA fail to recanalize and lack neurological improvement (15). In the study protocol described by Lilicrap et al., the authors hypothesize that individual plasmin potential, as measured by *in vitro* response to rt-PA, may serve as a biomarker of rt-PA response and patients with greater plasmin response are more likely to recanalize early (Lillicrap et al.). Their study will be based on historical samples from the Barcelona Stroke Thrombolysis Biobank, comprised of 350 pre-thrombolysis plasma samples from AIS patients who received serial transcranial Doppler measurements before and after thrombolysis. The primary outcome of the study will be time to recanalization detected by TCD (defined as TIBI ≥ 4). Results of this study may have important implications for the clinical practice: if association between proteolytic response to rt-PA and recanalization is confirmed, future clinical treatment may customize thrombolytic therapy to maximize outcomes and minimize adverse effects for individual patients.

Interestingly, our knowledge today is still limited about hemostasis alterations increasing the risk of stroke. In this Special Issue, informative case reports are published on inherited and acquired thrombophilic risk factors associated with stroke (Watanabe et al. and Huseynov et al.). Cases of cyclosporine A induced intracranial thrombotic complications and a systematic review of the literature is provided by Song et al., reviewing articles on cyclosporine A- related thrombotic events and summarizing features of clinical characteristics and neuroimaging findings in drug-induced cerebral venous thrombosis. Significant sex-differences in risk factors for transient ischemic attack (TIA) were found and published in this Special Issue by Wang et al. by screening a high-risk population of >230,000 residents in eastern China. This original work highlights that improving the understanding of sex differences in the prevalence of stroke risk factors is necessary to develop strategies to reduce stroke incidence and mortality. The research paper by Fang et al. aimed to investigate the association between intracranial atherosclerotic stenosis (ICAS) and the severity of white matter changes (WMC). Their work may improve our understanding regarding how the presence of ICAS would influence the progression of WMC, which could potentially provide future therapeutic opportunities for prevention.

In the past few years, new perspectives were opened in the field of thrombosis and vascular biology, when mechanical thrombectomy has permitted the histological analysis of retrieved thrombi of AIS patients. In depth characterization of the structure of ischemic stroke thrombi may serve as an important tool to provide useful insights on AIS pathomechanisms and treatment failure (16). In this Issue, two relevant papers were published assessing cellular, hemostatic and protein components of AIS thrombi. In their original research paper, Dargazanli et al. resolved the proteomes of cardioembolic and atherothrombotic cerebrovascular human thrombi and applied an artificial intelligence routine to examine protein signature between the two selected groups. Marta-Enguita et al. performed histological analysis of different hemostatic parameters including some key proteins, potentially determining thrombus stability that have not been investigated as yet: thrombin activatable fibrinolysis inhibitor (TAFI) and matrix metalloproteinase 10 (MMP-10). The authors report that histological composition and distribution of different thrombi hemostasis components have prognostic implications, and it could potentially have an impact on the strategies to guide personalized therapies for stroke patients.

Hemorrhagic stroke accounts for ~10–15% of all strokes and results in a higher rate of mortality as compared to ischemic strokes (17). In the IRONHEART study, published as a study protocol by Árokszállási et al.. and an original research paper by Orbán-Kálmándi et al. the authors aimed to test whether various hemostasis parameters may predict the outcome of non-traumatic intracerebral hemorrhage (ICH). Their results show that a modified clot lysis assay, that incorporates the effect of neutrophil extracellular traps correlated with the estimated bleeding volumes in patients with ICH and might serve as a useful tool to predict ICH outcomes. It is known that about 10% of intracerebral neoplastic lesions initially present as spontaneous ICH (18). In the work of Nawabi et al., the authors evaluated the potential of a machine learning-based prediction of etiology for acute ICHs based on quantitative radiomic image features extracted from initial non-contrast-enhanced tomography (NECT) brain scans. The quantitative evaluation of acute NECT images in a machine learning algorithm provided high discriminatory power in predicting non-neoplastic vs. neoplastic ICH, thus the authors suggest that using this approach in the clinical routine might improve patient care. Treatment options in ICH are often challenging due to the high mortality of this disorder (19). The paper by Apostolaki-Hansson et al. investigated whether the effect of oral anticoagulant (OAC) treatment reversal is beneficial in patients with ICH. They compared 90-day survival and outcome in patients with OAC-ICH who received OAC reversal therapy and those who did not using the database of The Swedish Stroke Register. Their results showed that patients receiving OAC-reversal treatment had an improved 90-day mortality outcome as compared to those not receiving treatment. Their results warrant larger studies to determine which patient groups are likely to benefit from reversal therapy. Another paper by Zhou et al. tested whether minimally invasive surgery or conservative treatment is more beneficial for patients with ICH. In their review paper, trial sequential analysis was applied on data from randomized trials to answer this question. They conclude that minimally invasive surgery is more effective than conservative treatment in patients with ICH in reducing morbidity and mortality. Repeating a clinical trial with similar devices, design and outcomes is unlikely to change current evidence.

We are confident that the papers in this Special Issue will be of interest and relevance to those involved in the experimental and clinical fields related to stroke and hemostasis. We hope that the published articles will provide ideas and inspiration to those dedicated to understanding the risk factors, pathophysiology and treatment outcomes of stroke in order to better diagnose, treat or prevent this devastating disorder in the future.

## Author Contributions

All authors listed have made a substantial, direct and intellectual contribution to the work, and approved it for publication.

## Conflict of Interest

The authors declare that the research was conducted in the absence of any commercial or financial relationships that could be construed as a potential conflict of interest.

## Publisher's Note

All claims expressed in this article are solely those of the authors and do not necessarily represent those of their affiliated organizations, or those of the publisher, the editors and the reviewers. Any product that may be evaluated in this article, or claim that may be made by its manufacturer, is not guaranteed or endorsed by the publisher.

## References

[1] Raskob GE Angchaisuksiri P Blanco AN Buller H Gallus A Hunt BJ. Thrombosis: a major contributor to global disease burden. Arterioscler Thromb Vasc Biol. (2014) 34:2363–71. 10.1161/ATVBAHA.114.304488 25304324

[2] Donkor ES. Stroke in the 21(st) century: a snapshot of the burden, epidemiology, and quality of life. Stroke Res Treat. (2018) 2018:3238165. 10.1155/2018/3238165 30598741 PMC6288566

[3] Bagoly Z Szegedi I Kalmandi R Toth NK Csiba L. Markers of coagulation and fibrinolysis predicting the outcome of acute ischemic stroke thrombolysis treatment: a review of the literature. Front Neurol. (2019) 10:513. 10.3389/fneur.2019.00513 31316444 PMC6611415

[4] Henderson SJ Weitz JI Kim PY. Fibrinolysis: strategies to enhance the treatment of acute ischemic stroke. J Thromb Haemost. (2018) 16:1932–40. 10.1111/jth.14215 29953716

[5] Thiebaut AM Gauberti M Ali C Martinez De Lizarrondo S Vivien D Yepes M. The role of plasminogen activators in stroke treatment: fibrinolysis and beyond. Lancet Neurol. (2018) 17:1121–32. 10.1016/S1474-4422(18)30323-5 30507392

[6] Jickling GC Liu D Stamova B Ander BP Zhan X Lu A. Hemorrhagic transformation after ischemic stroke in animals and humans. J Cereb Blood Flow Metab. (2014) 34:185–99. 10.1038/jcbfm.2013.203 24281743 PMC3915212

[7] Yaghi S Willey JZ Cucchiara B Goldstein JN Gonzales NR Khatri P. Treatment and outcome of hemorrhagic transformation after intravenous alteplase in acute ischemic stroke: a scientific statement for healthcare professionals from the American Heart Association/American Stroke Association. Stroke. (2017) 48:e343–61. 10.1161/STR.0000000000000152 29097489

[8] Gensicke H Al Sultan AS Strbian D Hametner C Zinkstok SM Moulin S. Intravenous thrombolysis and platelet count. Neurology. (2018) 90:e690–7. 10.1212/WNL.0000000000004982 29367438

[9] Powers WJ Rabinstein AA Ackerson T Adeoye OM Bambakidis NC Becker K. 2018 guidelines for the early management of patients with acute ischemic stroke: a guideline for healthcare professionals from the American Heart Association/American Stroke Association. Stroke. (2018) 49:e46–110. 10.1161/STR.0000000000000163 29367334

[10] Berge E Whiteley W Audebert H De Marchis GM Fonseca AC Padiglioni C. European Stroke Organisation (ESO) guidelines on intravenous thrombolysis for acute ischaemic stroke. Eur Stroke J. (2021) 6:I–LXII. 10.1177/2396987321989865 33817340 PMC7995316

[11] Scott RM Smith ER. Moyamoya disease and moyamoya syndrome. N Engl J Med. (2009) 360:1226–37. 10.1056/NEJMra0804622 19297575

[12] Emberson J Lees KR Lyden P Blackwell L Albers G Bluhmki E. Effect of treatment delay, age, and stroke severity on the effects of intravenous thrombolysis with alteplase for acute ischaemic stroke: a meta-analysis of individual patient data from randomised trials. Lancet. (2014) 384:1929–35. 10.1016/S0140-6736(14)60584-5 25106063 PMC4441266

[13] Niego B Medcalf RL. Plasmin-dependent modulation of the blood-brain barrier: a major consideration during tPA-induced thrombolysis? J Cereb Blood Flow Metab. (2014) 34:1283–96. 10.1038/jcbfm.2014.99 24896566 PMC4126105

[14] Warach SJ Dula AN Milling TJ Jr. Tenecteplase thrombolysis for acute ischemic stroke. Stroke. (2020) 51:3440–51. 10.1161/STROKEAHA.120.029749 33045929 PMC7606819

[15] Bhatia R Hill MD Shobha N Menon B Bal S Kochar P. Low rates of acute recanalization with intravenous recombinant tissue plasminogen activator in ischemic stroke: real-world experience and a call for action. Stroke. (2010) 41:2254–8. 10.1161/STROKEAHA.110.592535 20829513

[16] Brinjikji W Duffy S Burrows A Hacke W Liebeskind D Majoie C. Correlation of imaging and histopathology of thrombi in acute ischemic stroke with etiology and outcome: a systematic review. J Neurointerv Surg. (2017) 9:529–34. 10.1136/neurintsurg-2016-012391 27166383 PMC6697418

[17] Pinho J Costa AS Araujo JM Amorim JM Ferreira C. Intracerebral hemorrhage outcome: a comprehensive update. J Neurol Sci. (2019) 398:54–66. 10.1016/j.jns.2019.01.013 30682522

[18] Choi YS Rim TH Ahn SS Lee SK. Discrimination of tumorous intracerebral hemorrhage from benign causes using CT densitometry. Am J Neuroradiol. (2015) 36:886–92. 10.3174/ajnr.A4233 25634719 PMC7990598

[19] Dastur CK Yu W. Current management of spontaneous intracerebral haemorrhage. Stroke Vasc Neurol. (2017) 2:21–9. 10.1136/svn-2016-000047 28959487 PMC5435209

